# Diagnostic Performance of Artificial Intelligence-Based Computer-Aided Diagnosis for Breast Microcalcification on Mammography

**DOI:** 10.3390/diagnostics11081409

**Published:** 2021-08-04

**Authors:** Yoon Ah Do, Mijung Jang, Bo La Yun, Sung Ui Shin, Bohyoung Kim, Sun Mi Kim

**Affiliations:** 1 Department of Radiology, Seoul National University Bundang Hospital, Seoul National University College of Medicine, Seongnam 13620, Korea; 54440@snubh.org (Y.A.D.); mjjang74@gmail.com (M.J.); yunbola@gmail.com (B.L.Y.); shinsungui@gmail.com (S.U.S.); 2 Division of Biomedical Engineering, Hankuk University of Foreign Studies, Seoul 17035, Korea; bkim@hufs.ac.kr

**Keywords:** radiology, computer-aided diagnosis, breast cancer, artificial intelligence, mammography, diagnosis

## Abstract

The present study evaluated the diagnostic performance of artificial intelligence-based computer-aided diagnosis (AI-CAD) compared to that of dedicated breast radiologists in characterizing suspicious microcalcification on mammography. We retrospectively analyzed 435 unilateral mammographies from 420 patients (286 benign; 149 malignant) undergoing biopsy for suspicious microcalcification from June 2003 to November 2019. Commercial AI-CAD was applied to the mammography images, and malignancy scores were calculated. Diagnostic performance was compared between radiologists and AI-CAD using the area under the receiving operator characteristics curve (AUC). The AUCs of radiologists and AI-CAD were not significantly different (0.722 vs. 0.745, *p* = 0.393). The AUCs of the adjusted category were 0.726, 0.744, and 0.756 with cutoffs of 2%, 10%, and 38.03% for AI-CAD, respectively, which were all significantly higher than those for radiologists alone (all *p* < 0.05). None of the 27 cases downgraded to category 3 with a cutoff of 2% were confirmed as malignant on pathological analysis, suggesting that unnecessary biopsies could be avoided. Our findings suggest that the diagnostic performance of AI-CAD in characterizing suspicious microcalcification on mammography was similar to that of the radiologists, indicating that it may aid in making clinical decisions regarding the treatment of breast microcalcification.

## 1. Introduction

Screening mammography is the most common and most effective method for detecting early breast cancer, with a demonstrated effect in reducing breast cancer mortality [1,2]. However, the reported false-negative rate for screening mammography ranges from 10–30% [3,4,5]. Possible causes for missed breast cancers include dense parenchyma obscuring a lesion, perception error, and incorrect interpretation of a suspicious finding [6].

After the United States Food and Drug Administration approved the computer-aided detection system for mammography in 1998, it has been improved and has helped radiologists detect subtle features of malignancy by reducing the perception error [7]. In addition, it has increased the sensitivity of mammography by aiding in the detection of suspicious findings, such as microcalcifications, asymmetries, and masses regardless of breast density which is one of the important causes of false-negative cases [8,9,10].

In mammography screening, the presence of breast microcalcifications is one of the important findings of early breast cancer. Detection of microcalcification on mammography results in a diagnosis of malignancy in up to 0.3% of women screened. Previous computer-aided detection systems were able to detect microcalcifications on mammography [11,12] with improved sensitivity for calcification ranging from 80 to 100% [9,10,12]; however, they also increased false-positive rates [13]. The reported positive predictive value for malignancy of suspicious microcalcifications ranged between 15.9 and 90.6% [14,15]; thus, many benign microcalcifications were biopsied. Furthermore, previous computer-aided detection systems could not differentiate benign calcifications from suspicious microcalcifications suggesting early breast cancer such as ductal carcinoma in situ (DCIS).

Compared with traditional computer-aided detection systems, the next-generation systems provide both computer-aided detection and diagnosis with the development of deep learning algorithm by faster computers, greater storage capabilities, and availability of big data during the last decade [16]. For the diagnosis of microcalcifications on mammography, several deep learning models have also been applied, and their results were promising [17,18,19,20].

Recently developed artificial intelligence-based computer-aided diagnosis (AI-CAD) software predicts the possibility of malignancy (malignancy score) of the breast lesion, demonstrating higher diagnostic performance in breast cancer detection than radiologists [21,22]. However, to the best of our knowledge, no study has evaluated the performance of commercially available AI-CAD in predicting the malignancy risk of microcalcification on breast mammography.

Therefore, in the present study, we evaluated the diagnostic performance of AI-CAD compared to that of dedicated breast radiologists in diagnosing suspicious microcalcifications on mammography. Our findings indicated that AI-CAD and radiologists exhibited similar diagnostic performance in characterizing suspicious microcalcifications on mammography. Thus, AI-CAD may aid in making clinical decisions for treating breast microcalcifications. Furthermore, adjusting the radiologist’s final category according to the AI-CAD malignancy score also improved diagnostic performance, suggesting that AI-CAD support can help to reduce the number of unnecessary biopsies without missing cancers.

## 2. Materials and Methods

### 2.1. Ethics Statements

Our institutional review board approved this study (protocol code L-2020-544 and date of approval 5 May 2020). The requirement for informed consent was waived because of the retrospective nature of this study.

### 2.2. Patient Population

Between June 2003 and November 2019, 591 unilateral mammographies from 576 consecutive patients who underwent stereotactic breast biopsy or mammography-guided localization and excisional biopsy for suspicious microcalcification in our tertiary hospital were obtained. Before 2015, patients only underwent mammography-guided localization and excisional biopsy. Thus, there were two groups from 2015 to 2019, including those who underwent stereotactic breast biopsy and those who underwent mammography-guided localization and excisional biopsy. These 576 patients’ mammographies were applied AI-CAD.

**Figure 1:**
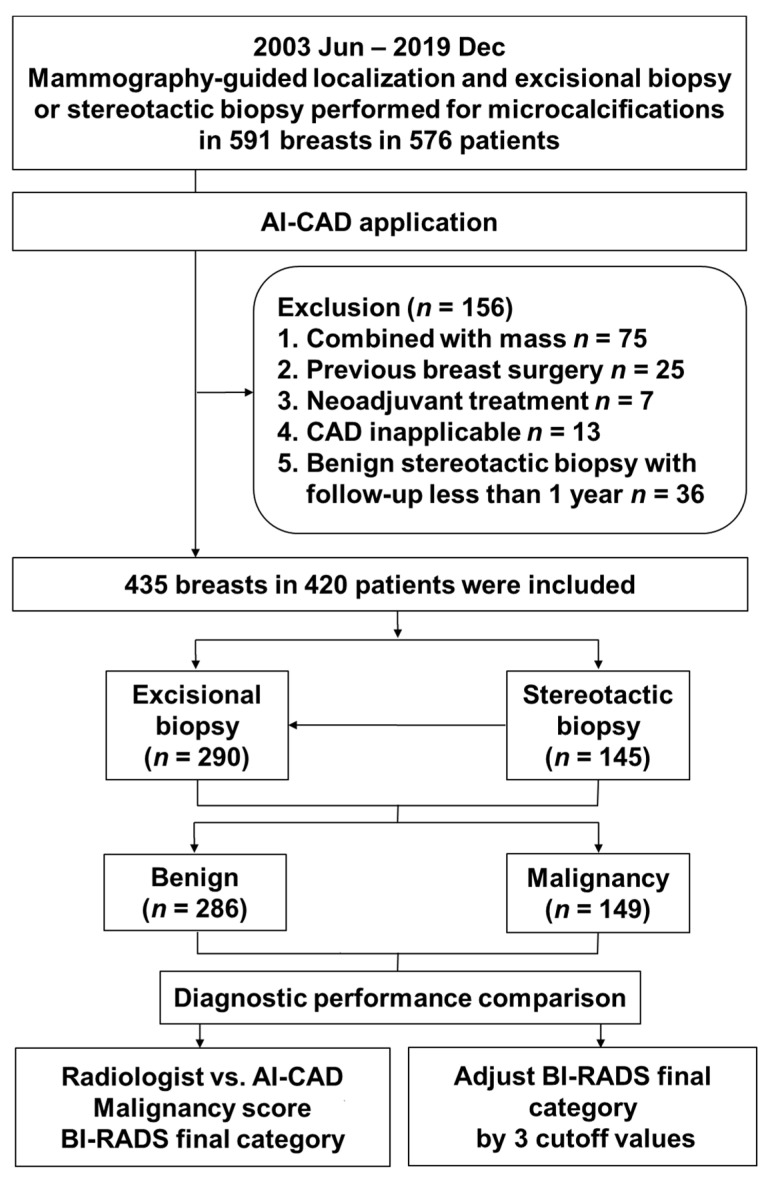
Flowchart of patient selection and AI-CAD applications. AI-CAD, artificial intelligence-based computer-aided diagnosis; BI-RADS: Breast Imaging Reporting and Data System.

Of these 591 mammographies, we first excluded cases with other confusing findings such as a mass lesion because, in such cases, the malignancy score predicted by AI-CAD is based not only on microcalcification but also on other findings in the same breast (75 patients). Second, patients who had undergone breast surgery at an outside hospital were excluded because residual microcalcifications and postoperative changes were not optimal for the evaluation of the characteristics of the microcalcification (25 patients). Third, patients who underwent preoperative treatment, such as neoadjuvant chemotherapy, were also excluded (7 patients). Fourth, because AI-CAD was only applicable to the bilateral routine four-view protocol for craniocaudal and mediolateral oblique views, patients in whom only the magnification view was obtained at biopsy were excluded (13 patients). Finally, among the patients who underwent a stereotactic biopsy and had benign biopsy findings, we excluded those who had been followed up for less than 1 year because there was a risk that the stereotactic biopsy did not represent the entire lesion (36 patients). Finally, 435 unilateral mammographies from 420 patients were included (Figure 1).

### 2.3. Electronic Medical Record Review

By reviewing the electronic medical records retrospectively, we extracted data related to patient age, symptoms, and risk factors for breast cancer, such as menopausal status, history of hormone replacement therapy (HRT) or breast cancer, or a family history of breast cancer. The final histopathology was determined as benign or malignant based on the pathological results of the biopsy specimen.

### 2.4. Image Interpretation

Three breast radiologists with 2, 14, and 16 years of experience in breast imaging, respectively, analyzed the characteristics of suspicious microcalcification without knowing the pathological results. The radiologist interpreted both routine mammography and magnification and compression views, similar to the scenario encountered in real practice. The radiologist measured the size of the suspicious microcalcification and determined its morphology, distribution, and the final category based on the fifth edition of the American College of Radiology BI-RADS revised in 2013 [14]. The breast parenchymal composition was also evaluated. One of the BI-RADS categories was assigned as the final category: category 3, probably benign; 4a, low suspicion of malignancy; 4b, intermediate suspicion of malignancy; 4c, moderate concern of malignancy; and 5, highly suggestive of malignancy [14]. The radiologist graded the mammography images with a probability of malignancy score, which was calculated on a 0–100 scale (0, definite non-cancer; 1–25, probably non-cancer; 26–50, possibly non-cancer; 51–75, possibly cancer; 76–99, probably cancer; and 100, definite cancer) [22,23].

### 2.5. Computer-Aided Diagnosis Software

**Figure 2:**
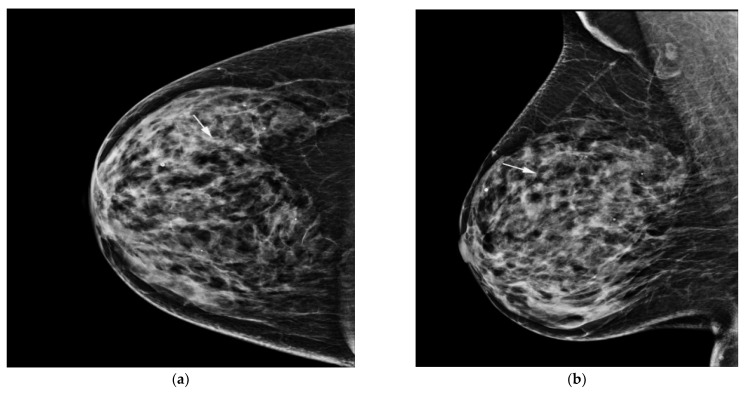

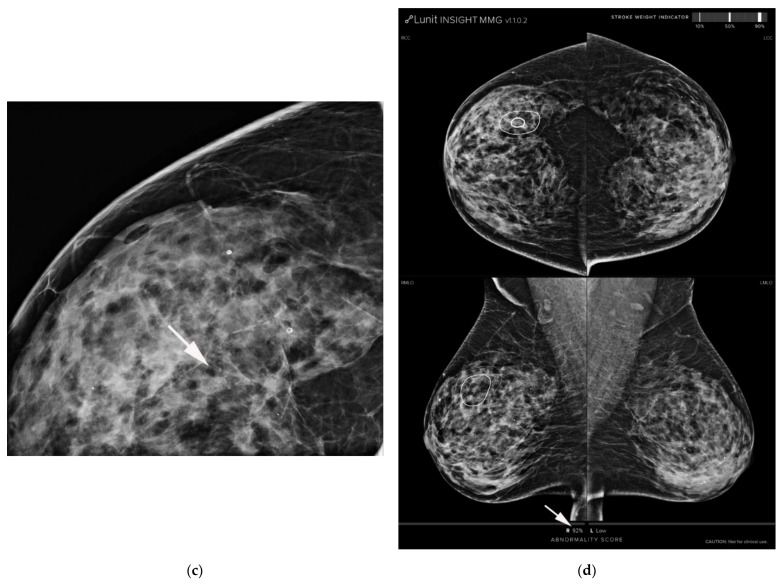
AI-CAD detected microcalcifications on screening mammography in a 64-year-old woman. (**a**,**b**) Right craniocaudal and mediolateral oblique routine mammography showed grouped microcalcifications (arrows). (**c**) The right craniocaudal magnification and compression view showed grouped amorphous microcalcifications (arrow). The radiologists categorized the microcalcifications as category 4a. (**d**) The malignancy rate determined by AI-CAD was 92% (arrow). The adjusted category was upgraded to 4b for all three cutoff values (2%, 10%, and 38.03%). Ductal carcinoma in situ was confirmed via stereotactic biopsy. Microinvasive carcinoma was confirmed via subsequent surgery. AI-CAD, artificial intelligence-based computer-aided diagnosis.

**Figure 3:**
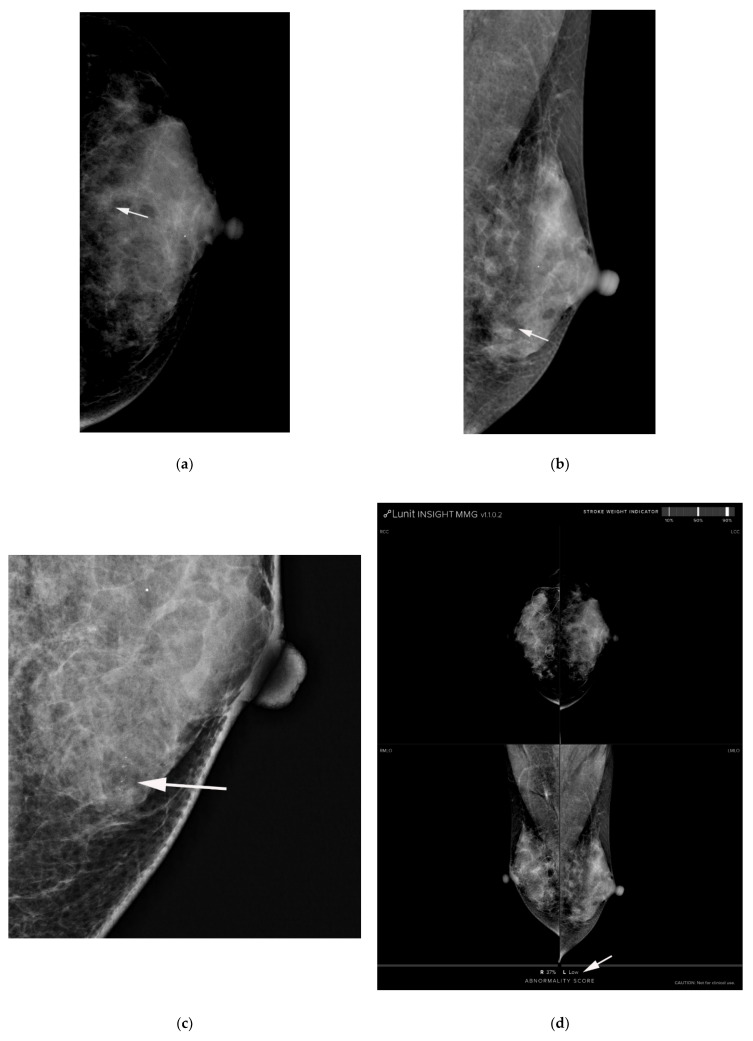
(**a**) AI-CAD did not detect microcalcifications on postoperative screening mammography in a 40-year-old woman. She had a history of breast-conserving surgery on her right breast. (**a**,**b**) Left craniocaudal and mediolateral oblique routine mammography showed grouped microcalcifications (arrows). (**c**) The left craniocaudal magnification and compression view showed grouped amorphous microcalcifications (arrow). The radiologists categorized the microcalcifications as category 4a. (**d**) The malignancy rated by AI-CAD was 1.21% (low, arrow). The adjusted category was downgraded to 3 for all three cutoff values (2%, 10%, and 38.03%). Fibrocystic changes with microcalcifications were confirmed by mammography-guided localization and excisional biopsy. AI-CAD, artificial intelligence-based computer-aided diagnosis.

We used diagnostic support software (Lunit insight MMG, Lunit, Seoul, Korea, available at https://insight.lunit.io, accessed 10 July 2021). Given the bilateral routine four-view protocol, AI-CAD provided a malignancy score of suspicious microcalcification in terms of a percentage (Figure 2 and Figure 3). If the predicted malignancy score of suspicious microcalcification was below 10%, AI-CAD offered a negative result. The cutoff was set to 10% because AI-CAD was developed to detect various findings suggesting malignancy as well as microcalcifications, and the best results were obtained using a cutoff of 10% [22]. Therefore, malignancy scores below the level of 10% were offered as raw data in the software database.

A breast radiologist with 19 years of experience in breast imaging confirmed the correlation of CAD and the radiologist’s marked area and biopsied area without blinding.

### 2.6. Adjustment of Radiologists’ Category Using AI-CAD Malignancy Score

The final category determined by radiologists was adjusted in consideration of the malignancy score predicted by AI-CAD. As a biopsy should be considered when the BI-RADS category is 4a (2–9% probability of malignancy) or higher, the category was upgraded by one step (i.e., category 3 to 4a, 4a to 4b, 4b to 4c, and 4c to 5) if the malignancy score predicted by AI-CAD was more than 2%. When the malignancy score was less than 2%, the category was downgraded by one step. Adjustment was conducted in the same manner with a cutoff of 10%, which was the baseline setting of the AI-CAD system, and a cutoff of 38.03%, which was the best cutoff we calculated. If the final category determined by radiologists was category 5, we did not upgrade the category.

### 2.7. Data and Statistical Analyses

The clinical, radiological, and pathological data of the 420 patients were collected for statistical analysis. Among the clinicopathological characteristics, categorical variables including presence of symptoms, malignancy risk factors, calcification morphology and distribution, and final assessment were compared between the benign and malignant cases using the chi-square test or Fisher’s exact test. In addition, continuous variables including age and calcification size were compared between the two groups using Student’s t-test.

Diagnostic performance was calculated for radiologists and AI-CAD using the final category determined by radiologists and malignancy score predicted by AI-CAD based on BI-RADS subcategorization: 3 (≤2% likelihood of malignancy), 4a (>2% but ≤10%), 4b (>10% but ≤50%), 4b (>50% but <95%), and 5 (≥95%) [14]. The malignancy scores predicted by AI-CAD and radiologists were compared using the area under the receiving operator characteristics curve (AUC). Based on the AUC results, the best cutoff of the malignancy score of AI-CAD for suspicious microcalcification was calculated. These statistical analyses were performed using MedCalc (MedCalc Software) and Stata (version 13 IC, StataCorp, College Station, TX, USA). A *p*-value < 0.05 was considered statistically significant.

## 3. Results

### 3.1. Patient and Lesion Characteristics

Of the 435 unilateral mammographies from 420 patients, mammography-guided localization and excisional biopsy were performed in 290, while stereotactic biopsy was performed in 145. Among the patients who underwent stereotactic biopsy, 71 patients subsequently underwent surgery because the pathological reports confirmed as atypia, DCIS (Figure 2), or invasive ductal carcinoma.

Overall, 286 cases were benign, and 149 were malignant based on the results of surgical excision (*n* = 361). Follow-up indicated stable, benign results in 74 cases. Baseline characteristics and those of suspicious microcalcifications in the benign and malignant groups are shown in Table 1. Patient age, breast density, and the extent of suspicious microcalcifications differed significantly between the benign and malignant groups: The malignant group was significantly older (age ± standard deviation (SD), 50.1 ± 9.6 vs. 47.8 ± 8.6 years, *p* = 0.01), exhibited calcifications with lower density (82.6% vs. 92%, *p* = 0.003), and had significantly larger extent of microcalcifications than the benign group (extent ± SD, 2.5 ± 2.0 cm vs. 1.8 ± 1.6, *p* < 0.001). There were no significant differences in the presence of symptoms or malignancy risk factors including the percentage of menopausal women, history of HRT, and personal history or family history of breast cancer. Regarding microcalcification morphology, the percentages of amorphous, coarse heterogeneous, fine pleomorphic, and fine linear calcifications were 68.2%, 17.5%, 12.9%, and 1.4%, respectively, in the benign group, and 28.9%, 16.1%, 43.0%, and 12.1%, respectively, in the malignant group (*p* < 0.001). As for the distribution of microcalcification, the percentages of grouped, linear, and segmental calcifications were 67.8%, 0.4%, and 12.6%, respectively, in the benign group and 53.7%, 3.4%, and 23.5%, respectively, in the malignant group (*p* < 0.001). Regarding the final BI-RADS category, the percentages of categories 4a, 4b, 4c, and 5 were 85.0%, 14.7%, 0.4%, and 0.0%, respectively, in the benign group, and 46.3%, 30.2%, 19.5%, and 4.0%, respectively, in the malignant group. There were significant differences in all categories between the two groups (all *p* ≤ 0.002).

### 3.2. Diagnostic Performance of Radiologists and AI-CAD

The AUC of the malignancy score determined using AI-CAD was slightly higher than that of the final category determined by radiologist using the malignancy score (0.745 vs. 0.722) and BI-RADS category (0.718 vs. 0.710), without a significant difference (*p* = 0.393 and 0.758) (Table 2). The calculated best cutoff of AI-CAD for detecting microcalcification with suspicious morphology was 38.03%.

### 3.3. Adjusted Radiologists’ Category Using AI-CAD Malignancy Score

**Figure 4:**
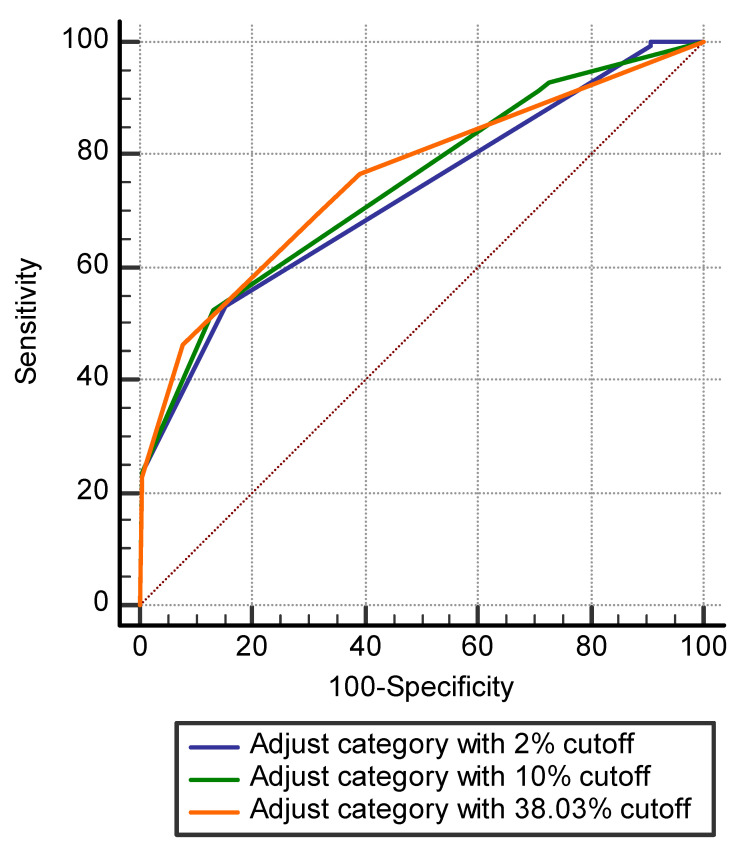
ROC curves of diagnostic performance of the adjusted radiologist’ category with cutoffs of 2%, 10%, and 38.03% of AI-CAD malignancy score. The AUCs of the adjusted category using malignancy score of AI-CAD with 2% cutoff was 0.726 (**blue line**), 10% were 0.744 (**green line**), and 38.03% were 0.756 (**orange line**), respectively.

Among 312 cases categorized as BI-RADS category 4a by radiologists, 27 (8.7%), 34 (10.9%), and 210 (67.3%) cases were downgraded to category 3 according to the AI-CAD malignancy score, with cutoffs of 2%, 10%, and 38.03%, respectively (Figure 3). The AUCs of the adjusted category using AI-CAD with those cutoffs were 0.726, 0.744, and 0.756, respectively, being slightly higher than those of radiologists’ (*p* < 0.05) (Table 3, Figure 4). None of the 27 cases downgraded to category 3 with a cutoff of 2% were confirmed as being malignant on pathological analysis.

## 4. Discussion

In this study, we compared the diagnostic performance of AI-CAD and dedicated breast radiologists in characterizing suspicious microcalcification on mammography. Our findings indicated that AI-CAD and radiologists provided similar malignancy scores for suspicious microcalcification. After adjusting the radiologists’ final category ratings according to three cutoff values (2%, 10%, and 38.03%) for the AI-CAD malignancy score, the AUCs of the adjusted category were higher than those of the radiologists’ category alone.

In up to 50% of all breast cancers, microcalcification is the only finding that can be detected on mammography [24], and about 93% of cases of DCIS involve microcalcification [25]. Thus, detection and interpretation of microcalcification are very important in the diagnosis of breast cancer. Computer-aided detection systems have increased the sensitivity of mammography, which has in turn increased the detection of microcalcifications [26,27]. Indeed, one study reported that using a computer-aided detection system to identify and mark clustered microcalcifications produced the most profound effect on radiologists’ performance in detecting all missed microcalcifications [26]. Missed microcalcifications have very inconspicuous characteristics owing either to their small size, obscuration by overlying fibroglandular tissues, or both, which cause them to be easily overlooked even by diligent radiologists. Baum et al. [27] evaluated the use of a traditional commercially available computer-aided detection system for full-field digital mammography. The authors reported a sensitivity value of 89% for the detection of microcalcifications and a rate of 0.35 false-positive marks per image for microcalcifications. However, a recent larger community-based study reported no improvement in screening performance with computer-aided detection, the cancer detection rate (4.1/1000), sensitivity (85.3% vs. 87.3%), and specificity (91.6% vs. 91.4%) were unchanged with and without computer-aided detection [28]. Another earlier study showed no change in cancer detection rate with and without computer-aided detection (4.2 vs. 4.15 per 1000), a non-significant increase in sensitivity (84% vs. 80.4%), but a significant decrease in specificity (87.2% vs. 90.2%), resulting in a nearly 20% increase in the biopsy rate [29].

Recently developed AI-CAD software attempts to go beyond simply detecting lesions, aiming to differentiate between benign and malignancy. A recent reader study reported that the performance of AI-CAD had an AUC of 0.938–0.970 and improved the diagnostic performance of the radiologist (AUC: 0.810–0.881) [22]. Another study reported higher AUC values when artificial intelligence support was used than when readers were unaided (0.89 vs. 0.87; *p* = 0.002) [30]. In this study the AUC values with AI-CAD (AUC: 0.718-756) are lower than those in the aforementioned studies because only microcalcifications lesions were included. Other applications of AI-CAD were the reduction of false-positive marking or false-negative findings [13,31] (Table 4).

Several studies using deep learning or convolutional neural network tried to characterize microcalcifications on mammography [17,18,19,20] (Table 4). Wang J et al. [17]’s deep learning model achieved a discriminative accuracy of 87.3% if microcalcifications were characterized alone. Cai H et al. [18] reported a sensitivity of 86.89% using the filtered deep features of convolutional neural network. Another recent study by Liu et al. [20] developed a combined deep learning model that incorporated mammography and clinical variables for predicting malignant breast microcalcifications in a BI-RADS 4 subset examined by radiologists. When they compared the performance of the combined model and breast radiologists in predicting the malignancy of breast microcalcifications, the combined model had achieved a diagnostic capability almost equivalent to that of a senior radiologist, and it significantly outperformed the junior radiologist (*p* = 0.029). In addition, improved performance (increased AUCs) was observed when junior radiologists were provided with the assistance of the deep learning model [20]. Lei C et al. [19] studied only BI-RADS category 4 microcalcifications with developing radiomic model, six radiomic features and the menopausal state were included in the radiomic nomogram, which the diagnostic performance for microcalcifications with an AUC of 0.80 in the validation cohort is significantly higher than radiologists (AUC 0.8 vs. 0.61 and 0.64, *p* < 0.05). Our result of the AUC for the AI-CAD malignancy score was slightly higher than that provided by the radiologist and was comparable to the previous reports, however, the difference was not statistically significant.

When breast radiologists analyze the characteristics of microcalcification, the additional magnification view may affect their assessment of the microcalcification category. Kim et al. [33] reported that the performance of radiologists improved when interpreting mammography with a magnification view. However, in our study, AI-CAD predicted the malignancy score using only mammography images obtained using a routine bilateral four-view protocol. Furthermore, considering that the radiologists who categorized suspicious microcalcifications were those with much experience in breast imaging, the performance of the radiologists may have been higher owing to these factors.

Schönenberger C et al. [32] reported that deep convolutional neural networks can be trained in order to successfully classify microcalcifications on mammograms according to the BI-RADS classification system with an accuracy of 99.5% for the BI-RADS 4 cohort, 99.6% for the BI-RADS 5 cohort, and 98.1% for the BI-RADS 4 and 5 cohort. In the present study, we used three different cutoff values (2%, 10%, and 38.03%) to adjust the final BI-RADS category determined by the radiologists. The cutoff of 10% is the suggested value from the developers of the AI-CAD [22], while those of 2% and 38.03% reflect BI-RADS category 3 and our best AUC result. Our findings indicated that the AUCs of the adjusted category were significantly higher than those of the radiologists alone (*p* < 0.05). Furthermore, it is interesting that none of the 27 cases downgraded to category 3 with a cutoff of 2% were confirmed as being malignant on pathological analysis, suggesting that AI-CAD assistance can help to avoid unnecessary biopsies.

There are several limitations to this study, including the use of data from a tertiary hospital, at which the prevalence of cancer is higher than that in the real community setting. Thus, there may have been selection bias, necessitating further studies using data obtained from community settings. Second, as previously mentioned, AI-CAD predicted the malignancy score based only on mammography images obtained using a routine bilateral four-view protocol. However, in real practice, when suspicious microcalcification is observed on routine mammography, additional magnification is frequently recommended. Therefore, the application of AI-CAD in the magnification view may further increase diagnostic performance. Third, when AI-CAD malignancy scores were below 10%, the area of interest was not marked, but cases with coexisting abnormalities other than microcalcifications had already been excluded. Fourth, we only compared diagnostic performance between AI-CAD and radiologists with much experience in breast imaging; thus, the performance of the radiologists may have been higher due to these factors, and our findings may have differed had less experienced radiologists participated. Additional studies with less experienced radiologists are needed in the future.

## 5. Conclusions

In conclusion, the present results demonstrated that the diagnostic performance of AI-CAD in characterizing suspicious microcalcification on mammography was similar to that of dedicated breast radiologists. The performance of AI-CAD was only based on routine four views of mammography without magnification view, which can be one of the advantages of AI-CAD. If the magnification view were incorporated, the performance of AI-CAD would be improved. In addition, adjusting the radiologist’s final category according to the AI-CAD malignancy score also improved diagnostic performance, suggesting that AI-CAD support can help to reduce the number of unnecessary biopsies without missing cancers. Thus, AI-CAD may aid in making clinical decisions for treating breast microcalcifications. This study only included BI-RADS category 4 or 5 microcalcifications with correlating pathological findings and compared the diagnostic performance with that of experienced radiologists. Further studies are needed in more realistic settings, including benign and possibly benign microcalcifications and radiologists with varying lengths of experience.

## Figures and Tables

**Table 1:** Baseline characteristics and characterization of microcalcification (*n* = 435).

Characteristic	Benign (*n* = 286)	Malignant (*n* = 149)	Total	*p*-Value
Age (Years), Mean ± SD	47.8 ± 8.6	50.1 ± 9.6	48.7 ± 9.0	0.01
Extent (cm)	1.8 ± 1.6	2.5 ± 2.0	2.0 ± 1.8	<0.001
Symptomatic	15 (5.2)	11 (7.4)	26 (6.0)	0.37
Cancer risk factor				
Menopausal	94 (32.9)	60 (40.3)	154 (35.4)	0.13
Personal history of HRT	25 (8.7)	19 (12.8)	44 (10.1)	0.19
History of breast cancer	41 (14.3)	16 (10.7)	57 (13.1)	0.29
Family history of breast cancer	21 (7.3)	19 (12.8)	40 (9.2)	0.06
Dense breast	263 (92.0)	123 (82.6)	386 (88.7)	0.003
Calcification morphology				<0.001
Amorphous	195 (68.2)	43 (28.9)	238 (54.7)	<0.001
Coarse heterogeneous	50 (17.5)	24 (16.1)	74 (17.0)	0.72
Fine pleomorphic	37 (12.9)	64 (43.0)	101 (23.2)	<0.001
Fine linear	4 (1.4)	18 (12.1)	22 (5.1)	<0.001
Calcification distribution				0.001
Diffuse	1 (0.4)	1 (0.7)	2 (0.5)	1
Regional	54 (18.9)	28 (18.8)	82 (18.9)	0.98
Grouped	194 (67.8)	80 (53.7)	274 (63.0)	0.004
Linear	1 (0.4)	5 (3.4)	6 (1.4)	0.02
Segmental	36 (12.6)	35 (23.5)	71 (16.3)	0.004
Final assessment (category)				<0.001
Category 4a	243 (85.0)	69 (46.3)	312 (71.7)	<0.001
Category 4b	42 (14.7)	45 (30.2)	87 (20.0)	<0.001
Category 4c	1 (0.4)	29 (19.5)	30 (6.9)	<0.001
Category 5	0 (0.0)	6 (4.0)	6 (1.4)	0.002

Note: Data are presented as the number of patients and percentage in parentheses, unless specified otherwise. HRT, hormone replacement therapy; SD, standard deviation.

**Table 2:** Diagnostic performance of radiologist and AI-CAD.

Performer	AUC (95% CI)	*p*-Value	Cutoff Value	Sensitivity (%)	Specificity (%)
Malignancy score					
Radiologist	0.722 (0.677–0.763)	0.393	43.5	54.4	89.2
AI-CAD	0.745 (0.701–0.785)		38.03	69.1	69.01
BI-RADS Category					
Radiologist	0.710 (0.665–0.752)	0.758	Category 4a	53.7	85
AI-CAD	0.718 (0.673–0.760)		Category 4b	59.1	78.2

AI-CAD, artificial intelligence-based computer-aided diagnosis; AUC, area under the receiving operator characteristic curve; BI-RADS: Breast Imaging Reporting and Data System; CI, confidence interval.

**Table 3:** Area under the receiving operator characteristic curve for diagnostic performance of adjusted radiologists’ category using AI-CAD malignancy score.

Category	AUC (95% CI)	*p*-Value	Cutoff Value	Sensitivity (%)	Specificity (%)
Radiologist	0.710 (0.665–0.752)		Category 4a	53.7	85
Adjusted at 2% cutoff	0.726 (0.682–0.768)	0.026	Category 4b	53	85
Adjusted at 10% cutoff	0.744 (0.701–0.785)	0.014	Category 4b	53.4	87.1
Adjusted at 38.03% cutoff	0.756 (0.713–0.796)	0.013	Category 4b	46.33	92.3

Note: Each *p*-value was compared with the radiologist’s result. AI-CAD, artificial intelligence-based computer-aided diagnosis; AUC, area under the receiving operator characteristic curve; CI, confidence interval.

**Table 4:** Diagnostic performances for mammography with artificial intelligence.

Study	Purpose	AI Method	Result
Mayo et al. [13]	Determine to reduce false positive per image with AI-CAD	Deep learning	Significant reductions in false marks with AI-CAD; calcifications (83%), mass (56%) with no reduction in sensitivity
Wang et al. [17]	Improve the diagnostic accuracy of microcalcifications with deep learning-based models	Deep learning	Accuracy was increased by adopting a combinatorial approach to detect microcalcifications and masses simultaneously.
Cai et al. [18]	Characterize the calcifications by descriptors obtained from deep learning and handcrafted descriptors	CNN	Classification precision of 89.32% and sensitivity of 86.89% using the filtered deep features in microcalcifications
Lei et al. [19]	Development of a radiomic model for diagnosis of BI-RADS category 4 calcifications	LASSO algorithm	The identification ability of the radiomic nomogram including six radiomic features and the menopausal state was strong with an AUC of 0.80.
Liu et al. [20]	Investigate deep learning in predicting malignancy of BI-RADS category 4 microcalcifications	Deep learning	The combined model achieved non-inferior performance as senior radiologists and outperformed junior radiologists.
Kim et al. [22]	Evaluate whether the AI algorithm can improve accuracy of breast cancer diagnosis	Deep CNN	AUC of AI (0.940) vs. average of radiologists (0.810) and AUC of radiologists improved with AI (0.801–0.881).
Rodríguez-Ruiz et al. [30]	Compare the performances of radiologists with and without AI system	Deep CNN	AUC with AI (0.89) higher than without AI (0.87) and sensitivity with AI (86%) higher than without AI (83%)
Watanabe et al. [31]	Determines the efficacy of AI-CAD in improving radiologists’ sensitivity in detecting originally missed cancers.	Deep learning	Statistically significant improvement in radiologists’ accuracy and sensitivity for detection of originally missed cancers
Schönenberger et al. [32]	Investigate the potential of a deep convolutional neural network to accurately classify microcalcifications	Deep CNN	The accuracy was 39.0% for the BI-RADS 4 cohort, 80.9% for BI-RADS 5 cohort, and 76.6% for BI-RADS 4 + 5 cohort.

AI-CAD, artificial intelligence-based computer-aided diagnosis; AUC, area under the receiving operator characteristic curve; CNN, convolutional neural network; LASSO, least absolute shrinkage and selection operator.

## Data Availability

All data generated and analyzed during this study are included in this published article. Raw data supporting the findings of this study are available from the corresponding author on request.
